# Periodontal and Peri-Implant Health Status in Traditional vs. Heat-Not-Burn Tobacco and Electronic Cigarettes Smokers: A Systematic Review

**DOI:** 10.3390/dj10060103

**Published:** 2022-06-08

**Authors:** Francesco D’Ambrosio, Massimo Pisano, Alessandra Amato, Alfredo Iandolo, Mario Caggiano, Stefano Martina

**Affiliations:** Department of Medicine, Surgery and Dentistry “Schola Medica Salernitana”, University of Salerno, Via S. Allende, 84081 Baronissi, Italy; pisano.studio@virgilio.it (M.P.); aamato@unisa.it (A.A.); aiandolo@unisa.it (A.I.); macaggiano@unisa.it (M.C.); smartina@unisa.it (S.M.)

**Keywords:** tobacco, cigarette smoking, smokers, electronic cigarettes, electronic nicotine delivery system, E-Cigs, periodontal disease, periodontitis, peri-implant disease, peri-implantitis

## Abstract

The aim of the present systematic review was to evaluate and possibly differentiate the effects of traditional cigarettes, heat-not-burn tobacco, and electronic cigarettes on periodontal and peri-implant health status. Electronic cigarettes and heat-not-burn tobacco have become very popular in recent years and have been proposed to consumers as a safer alternative to conventional tobacco smoke, although their effect on periodontal and peri-implant health remains unclear. The study protocol was developed according to PRISMA guidelines, and the focus question was formulated according to the PICO strategy. A literature search was conducted across PubMed/MEDLINE and the COCHRANE library from 2003 to April 2022. From the 1935 titles initially identified, 18 articles were finally included in the study and extracted data were qualitatively synthesized. It may be carefully concluded that e-cigarettes may cause attenuated clinical inflammatory signs of periodontitis and, hypothetically, of peri-implantitis when compared to conventional tobacco smoke. Both alternative smoking products, containing nicotine, may likewise exert negative effects on periodontal and peri-implant health, as demonstrated by in vitro studies. Further investigations are needed to assess the impact of electronic cigarettes and heat-not-burn tobacco products on periodontal and peri-implant health status.

## 1. Introduction

Periodontitis is a chronic inflammatory disease of bacterial etiology, affecting and progressively destroying the tissues supporting the teeth and eventually leading to bone and tooth loss [1,2,3,4,5]. Analogously, peri-implantitis is an inflammatory lesion of the soft tissues surrounding an endosseous implant, leading to progressive peri-implant bone loss until implant failure [1,2,3,4,5]. Both periodontal and peri-implant diseases most frequently occur in adulthood and have been found or proposed to be associated, mainly through systemic inflammation, to a variety of systemic inflammatory disorders, including cardiovascular and pulmonary ones, diabetes, obesity, preterm birth, Alzheimer disease, and benign and solid malignant tumors [6,7,8,9,10,11].

Although periodontitis and peri-implantitis are both bacterial infections involving dental biofilm, suspected periodontal pathogens, individual age at disease onset, periodontitis severity, and rate of progression are all crucially influenced by well-known systemic factors, including inflammatory disorders—most importantly diabetes [6,7,8,9,10]—and neoplasms [11], as well as unhealthy habits [12], especially smoking [13,14].

Electronic cigarettes (E-Cigs) were introduced in the United States of America in 2006 and, since 2014, have become the most widespread tobacco product among young people between 18 and 25 years of age as a pastime [15], as well as among adults as an alternative to regular tobacco cigarettes and to quit smoking [16], similarly to the heat-not-burn (HNB) tobacco products that are becoming a new global trend [17].

E-cigarettes are small handheld devices containing a battery heating a solution and producing an aerosol. Usually, the liquid contains a mixture of substances such as nicotine, humectants, and flavoring chemical agents [18,19]. However, traces of heavy metals, such as aluminum, arsenic, nickel, and other contaminants very dangerous for human health have been found [20].Accordingly, Gaur and Agnihotri demonstrated that E-Cig use, also referred to as “vaping”, is not a safe alternative to normal tobacco cigarettes because the vaping liquid itself contains elements and toxic heavy metals predisposing the user to chronic pathological conditions [21].

Moreover, Moreover, Rahlo et al. and Yang et al. concluded that although electronic cigarette users may somehow be considered healthier than conventional cigarette smokers, they are still predisposed to the development of oral mucosal lesions and to dental and periodontal damage compared to non-smokers [22]. Figueredo et al., based on limited data available, reported that e-cigarettes have an unhealthy effect on periodontal health [23]. Similarly, Jeong et al., evaluating periodontal health status in 13,551 conventional and E-Cig smokers, concluded that electronic cigarette vaping and conventional cigarette smoking were both risk factors for periodontal diseases [24].

Furthermore, results from in vitro studies showed that E-Cig use is capable of altering myofibroblasts differentiation, causing DNA damage, inducing oxidative stress, and increasing inflammatory cytokines in human gingiva and periodontal ligament fibroblasts [25]. A recent study has also shown that the concentrations of both albumin and uric acid detectable in the whole saliva differ between smokers and non-smokers, being reduced in non-smokers [26].

Tobacco heating systems instead employ a particular heating method, reaching lower temperatures (240–350 °C) compared to traditional tobacco (>600 °C) [26], thus avoiding combustion. To our knowledge, only one study, not considering self-reported periodontitis, has presented provisional results describing more favorable periodontal treatment outcomes in HNB compared to conventional tobacco smokers [27]; however, it has been reported that HNB tobacco may potentially enhance oral epithelial cell proliferation [28].

Considering that both E-Cigs and HNB tobacco are proposed to consumers as less harmful compared to traditional cigarettes and as a safer alternative to conventional tobacco [26], a comparison of their effect on periodontal and peri-implant health status, while also evaluating former or current traditional tobacco smokers, may be especially relevant, both in the prevention and treatment planning of periodontal and peri-implant diseases. Therefore, the aim of the present systematic review was to evaluate and possibly differentiate the effects of conventional cigarettes, electronic cigarettes, and heat-not-burn tobacco products on periodontal and peri-implant status.

## 2. Materials and Methods

### 2.1. Protocol Development

The study protocol was developed according to the PRISMA (Preferred Reporting Items for Systematic Review and Meta-Analyses) guidelines [29,30]. The research question was formulated according to the PICO (Population, Intervention, control or Comparison, Outcome) strategy [31]:

P (Population): Smokers;

I (Intervention): Electronic cigarettes and heat-not-burn tobacco systems;

C (Comparison): Non-smokers, ex-smokers, and tobacco cigarette smokers;

O (Outcome): Clinical, radiographic, and inflammatory periodontal and peri-implant tissue parameters.

The clinical question in “PICO” format was: Is there a significant difference in clinical, radiographic and inflammatory parameters of periodontal and peri-implant tissues from e-cigarette users and Heat-Not-Burn tobacco when compared to tobacco users and non-smoking subjects?

### 2.2. Search Strategy and Study Selection

A literature search was independently conducted by two reviewers (F.D.A., S.M.) through PubMed/MEDLINE and the COCHRANE library. Only articles published after 1st January 2003 (when the electronic cigarette was introduced) till 24th April 2022, in the English language, were included.

A combination of the following keywords was employed for the electronic search:

Periodontal disease OR periodontitis OR peri-implant disease OR peri-implantitis OR dental implant OR implant loss OR plaque index OR gingival index OR bleeding on probing OR probing depth OR tooth loss OR missing teeth OR marginal bone level OR IL-1b OR IL-8 OR IL-6 OR TNF-a OR MMP-1 OR MMP-8 OR IFN-y OR IL-4 OR IL-9 OR IL-10 OR IL-13 OR OPG OR RANK-LAND OR e-cigarette OR vaping cigarette OR electronic cigarette OR electronic nicotine delivery system OR Heat-Not-Burn Tobacco OR vape OR vaping.

Articles were included if they were published in the English language, after 1st January 2003, and described clinical trials and/or observational studies assessing clinical and/or radiographic periodontal and/or peri-implant parameters. Records were excluded if study participants were <18 years old and in case of missing data concerning clinical and/or radiographic periodontal and/or peri-implant parameters; systemic and narrative reviews and preclinical studies were also not considered in the current study.

### 2.3. Data Extraction and Synthesis

Extracted data concerned: author(s) and year of publication; study design; total number, mean age, gender ratio and smoking habits of participants; periodontal status, number of implants, and periodontal and peri-implant clinical, radiographic and crevicular parameters, including clinical attachment loss (CAL) probing depth (PD), bleeding on probing (BOP), plaque index (PI), gingival index (GI), marginal bone levels (MBL), cytokines profile and periodontal treatment.

### 2.4. Risk of Bias Assessment

The risk of bias of the non-randomized clinical trials was evaluated through the ROBINS-I (Risk Of Bias In Non-randomized Studies of Interventions) tool, considering biases due to confounding, selection of participants, classification of interventions, deviations from intended interventions, missing data, selection of the reported result and biases in the measurement of outcomes and biases due to [32].

Risk assessment was conducted according to the following criteria [32]:

- Low risk of bias: The study is judged to be at low risk of bias for all domains;
- Moderate risk of bias: The study is judged to be at low or moderate risk of bias for all domains;
- Serious risk of bias: The study is judged to be at serious risk of bias in at least one domain, but not at critical risk of bias in any domain;
- Critical risk of bias: The study is judged to be at critical risk of bias in at least one domain.

## 3. Results

### 3.1. Search Strategy and Study Selection

Electronic search and study selection were independently conducted by two reviewers (F.D.A., S.M.) and disagreements were discussed and solved.

Titles obtained through the electronic search were screened to eliminate duplicates. Abstracts of the pertinent records were screened according to eligibility criteria and related full texts were examined. In total, 1935 records were retrieved from PubMed/MEDLINE (1793 articles) and the COCHRANE library (142 articles). A total of 98 titles were removed because duplicates and 1796 titles were excluded as they were not pertinent to the topic of the present review; therefore, 41 articles were considered eligible and full texts were obtained. After the full-text evaluation according to the selection criteria, 23 studies were excluded (Table 1), specifically because: 11 did not describe clinical/radiographic parameters nor cytokine profile (11); 2 exclusively investigated the relationship between e-cigarettes and oral microbiome and 1 between e-cigarettes and alcohol, respectively; 2 studies involved former tobacco smokers who started using E-Cigs; 1 study assessed the effect of antimicrobial photodynamic therapy (aPDT) in E-Cigs smokers; and 6 articles were systematic reviews of the literature.

**Figure 1:**
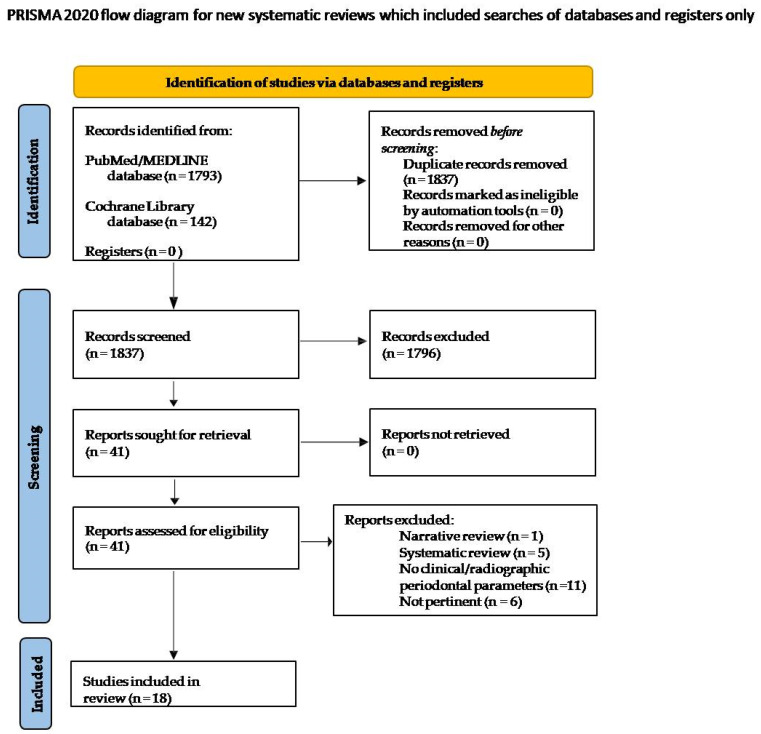
Study selection flowchart.

Finally, 18 articles were included in the present systematic review (Figure 1).

### 3.2. Study Characteristics

Table 2 illustrates the characteristics of the 18 included studies, concerning source, study design, aim(s), participants, periodontal and peri-implant parameters considered, main results and conclusions.

All included studies evaluated periodontal parameters and in 7 out of 18 peri-implants were also assessed. Extracted and analyzed periodontal and peri-implant parameters concerned traditional and electronic cigarettes smokers, whilst no study analyzed findings from HNB tobacco smokers; results from waterpipe smokers were also reported in the synthesis of the results and briefly discussed, although not relevant for the present study, since they could not be extrapolated from data comparison among study groups.

Due to the heterogeneity of the included studies and to the lack of randomized controlled trials, it was not possible to conduct a meta-analysis.

### 3.3. Data Extraction and Synthesis

Detailed findings related to periodontal clinical and radiographic, as well as inflammatory parameters, are synthesized in Table 3 and in Table 4, respectively; Table 5 describes peri-implant clinical and radiographic parameters Table 6 reports inflammatory ones.

### 3.4. Quality Assessment of the Included Studies

The risk of bias of the studies included in the present systematic review is detailed in Table 7.

## 4. Discussion

Electronic cigarettes and Heat-Not-Burn tobacco products are becoming very popular among the population, especially as many people think they are less harmful than conventional tobacco; therefore, the aim of the present systematic review was to evaluate the effects of electronic cigarettes and heat-not-burn tobacco products on periodontal and peri-implant status compared to traditional tobacco use. Unfortunately, data from clinical studies on Heat-Not-Burn tobacco systems were lacking and retrieved data on electronic cigarettes were heterogeneous, thus precluding the possibility of conducting a meta-analysis and, therefore, representing the main limitation of the study. Analyzed and qualitatively synthesized data are discussed below.

### 4.1. Clinical Periodontal and Peri-Implant Parameters in Traditional vs. HNB and E-Cigs Smokers

Jeong et al. suggested that vaping may not be a safe alternative to cigarette smoke; indeed, periodontitis was more prevalent in E-Cigs and CS than NS [26]; this finding may be considered especially relevant since this study, based on community periodontal status (CPI), included more participants (5715 males and 7836 females) than other studies included in this review [26].

A typical feature of periodontal disease associated with tobacco smoking is a greater destruction of the supporting tissues of the teeth with clinical attachment loss [67]. Many studies, reported in this review, agreed with this; in fact, Mokeem et al., BinShabaib et al., Ibraheem et al. and Fangxi Xu et al. described that CS had the worst CAL values compared to NS [50,54,58,65]. Aldakheel et al. also came to the same conclusion, reporting higher CAL values in CS, E-Cigs and NS with periodontitis compared to NS without periodontitis [57]. Nevertheless, Ibraheem et al. found similar CAL values between CS and E-Cigs [58]; consistently, Vohra et al. and Javed et al. found no difference in CAL values between CS, E-Cigs and NS [55,56]. Mokeem et al. and BinShabaib et al. found no statistically significant differences in CAL between NS and E-Cigs [50,54]. Similar results were also reported by Al-Hamoudi before and after SRP [61].

Regarding Bleeding on Probing, which is a clinical sign of periodontal and peri-implant tissues inflammation [1,2,8,9,10], several studies included in the present systematic review [50,53,54,56,59,60,62,64] showed an increased BoP in NS compared to CS and E-Cigs, with no differences, instead, between CS and E-Cigs [50,54]. These findings suggest that nicotine-containing e-cigarettes may cause vasoconstriction within both outer periodontal and peri-implant tissues, similarly to traditional tobacco products [68], although such an effect may not be as strong as that that related to tobacco products. Tatullo et al. found that BoP decreased over a 4-month period in e-cigarette smokers who were former smokers, even if they had been smoking for more than 10 years [47]; nonetheless, a few authors did not find statistically significantly differences between smokers and those who had never smoked [51,58,64].

Regarding plaque accumulation, many authors reported higher PI values in CS and E-Cigs compared to NS [53,58,59,64]. In more detail, Vohra et al. reported that CS had the worst PI values compared to E-Cigs and NS [55]; conversely, AlJasser et al. found that NS had worse plaque conditions than E-Cigs and CS [65]. Mokeem et al. also found higher, although not significantly different, PI values around natural teeth in CS than E-Cigs and in E-Cigs than in, otherwise ArRejaie et al. did not find any differences in PI around dental implants between CS and E-Cigs [50,60]. Aldakeel et al. instead found that CS, E-Cigs and NS with periodontitis had a statistically significantly higher PI compared to NS, CS and E-Cigs without periodontitis [57]. However, nicotine appears to induce proliferation of suspected periodontal pathogens, as *A. actinomycetemcomitans* and *P. gingivalis*, which were more frequently detected in CS and E-Cigs gingival biofilm compared to NS smokers with periodontitis plaque [57]. Al-Aali et al., Sinha et al., BinShabaib et al. and Karaaslan et al. found no statistically significant differences in PI values between E-Cigs and NS [51,52,54,64]. Al-Hamoudi et al. also found no differences among PI in E-Cigs and NS at baseline, but after a 3-month follow-up, significant reductions in PI in NS were described [61].

Regarding the Probing Depth, several authors described overall worse PD in Cs and E-Cigs compared to NS [51,53,54,55,56,58,60,62,63], despite the fact that E-Cigs showed less clinical signs related to periodontal and peri-implant inflammation and disruption to CS [55]. Al Qahtani et al. found significantly lower PD values in E-Cigs compared to CS and WS [53], supporting the hypothesis that cigarette smoke may be responsible for periodontal tissue destruction and cell death and may increase the production of matrix metalloproteinases involved in the inflammatory process [55]. Karaslaan et al. [52], as well as AlDakheel et al. [57], did not find significant differences in PD values among CS, E-Cigs and NS with and without periodontitis [57]. Although many authors found no differences in PD values among NS and E-Cigs [50,54,59], Alhamoudi et al. [61], who similarly reported similar findings at baseline between NS and E-Cigs, described, following mechanical periodontal treatment, a significant PD reduction in NS but not in E-Cigs.

As for GI, it was found to be significantly higher in E-Cigs compared to CS [52] and in CS, E-Cigs, and NS with periodontitis compared to NS without periodontitis (*p* < 0.001); no statistically significant difference among CS, E-Cigs, and NS with periodontitis was observed, but instead for GI [57]. Noteworthy, GI was reported to be significantly higher in NS than E-Cigs at baseline, but at the 3-month follow-up, a statistically significant improvement was observed in NS [61] but not in E-Cigs.

Many authors did not find statistically significant differences for MT among CS, E-Cigs and NS, probably because the follow–up period considered in the studies was too short [54,55,56,59,60].

### 4.2. Radiographic Periodontal and Peri-Implant Parameters in Traditional vs. HNB and E-Cigs Smokers

Marginal bone loss was generally higher in CS compared to NS [50,53,54,58,60].

Conversely, Vohra et al. and Javed et al. found no differences in MBL among NS, E-Cigs and CS, although Javed et al. found significantly higher MBL in older (>65 years) smokers and nonsmokers compared to younger (<45 years) ones [55,56]. No statistically significant difference in MBL among CS, E-Cigs and NS with periodontitis was found by Aldakeel et al., revealing, however, a greater MBL in periodontal smokers (CS and E-Cigs) and nonsmokers compared to NS without periodontitis, as expected [57]. No differences in MBL were found by Binshabib et al. and Mokeem et al. between NS and E-Cigs and by Al Hamoudi et al., both at baseline and after periodontal treatment [50], although opposite results were reported instead by other authors [51,58,63].

### 4.3. Crevicular Inflammatory Periodontal and Peri-Implant Parameters in Traditional vs. HNB and E-Cigs Smokers

Pro-inflammatory biomarkers have also been analyzed by many authors. In particular, Interleukin IL-1 β and Tumor Necrosis Factor-alpha (TNF-α), detectable in PISF (peri-implant sulcular fluid), may be considered as biomarkers for both periodontal and peri-implant diseases diagnosis and prognosis [51,62]. PISF levels were generally higher in smokers compared to nonsmokers [53,60,62,64] and in E-Cigs compared to NS [51,63]. Pro-inflammatory cytokines, such as TNF-α, IL-6 and IL-1β, secreted by activated macrophages in response to bacterial lipopolysaccharide [69,70], may potentially play a crucial role in periodontal and peri-implant tissue inflammation and destruction [53,60,62], stimulating osteoclastogenesis, osteoclasts activation with subsequent bone resorption, and inducing fibroblasts apoptosis [71,72], and have been found increased in saliva and in GCF of CS and WS and E-Cigs compared to NS [50,51,55,56,73]. Al-Hamoudi et al. found, after mechanical periodontal treatment, higher crevicular IL-4, IL-10, IL-11 and IL-13 levels in E-Cigs with moderate chronic periodontitis compared to NS with moderate chronic periodontitis, assuming that nicotine may compromise periodontal healing [61]. Bin Shabaib et al. and Mokeem et al. reported significantly higher IL-1β, IL-6 and TNF-a levels in CS compared to E-Cigs and NS, while no differences were found among E-Cigs and NS, probably because, in this study, E-Cigs participants were vaping for a relatively short duration [50,54]. Additionally, ArRejaie et al. obtained similar results comparing CS, E-Cigs and NS, but he also found statistically significant differences between E-Cigs and NS for IL-1b, where IL-b levels were higher in E-Cigs than NS [60]; accordingly, Al–Ali et al. and Sinha et al. obtained the same results between E-Cigs and NS [51,63]. Al Quatani et al. found similar IL-1b, IL-6 and TNF-a levels among CS, WS and E-Cigs, all significantly higher than those from NS [53]. Similar findings were also reported for crevicular TNF-a values by other authors with a general reduction after periodontal treatment [64].

RANKL (receptor activator of nuclear factor-kappaB ligand), RANK (receptor activator for nuclear factor-kappaB) and OPG (osteoprotegerin) mainly regulate osteoclast activity [72,74]. Coherently, Bostanci et al. described periodontal subjects who showed a significantly higher RANKL/OPG ratio compared to periodontally healthy ones [6,71,72,75,76]. Ibraham et al. demonstrated that crevicular RANKL and OPG levels were higher in CS and E-Cigs compared to NS [58].

CS and E-Cigs had the same adverse effects on oxidative stress markers and inflammatory cytokines, as demonstrated by significantly higher Glutathione peroxidase (GSH-Px) levels detected in NS was compared to CS and E-Cigs; however, no significant difference between CS and E-Cigs was found [52,77]. Higher levels of GSH-Px, protecting tissues from oxidative stress, has also been found in subjects with periodontitis [52].

Moreover, nicotine increases the accumulation in periodontal and peri-implant tissues of Advanced Glycation and Products (AGEs), along with their receptors (RAGEs), which have been associated with the formation of ROS (reactive oxygen species), inducing, in turn, oxidative stress and metabolic changes [27,28] within tissues. Currently analyzed data on MMP-8 and MMP-9, specifically activated by ROS [78], revealed significantly higher levels in CS and E-Cigs compared to NS, once more supporting the contributing role of nicotine to periodontal and peri-implant tissues destruction [54,60,64].

Furthermore, cotinine, which is a nicotinic metabolite that remains in saliva and crevicular fluid for up to 1 week after using nicotine-containing products, has been found in higher concentrations in CS, WS and E-Cigs PISF compared to NS [62], as expected, although no significant differences were found by Alquantani et al. and Mookem et al. among individuals using nicotinic products [50,62]. Conversely, Fangxi Xu et al. described higher crevicular cotinine levels in CS compared to E-Cigs.

Further studies are needed to highlight the impact of electronic cigarettes and Heat-Not-Burn tobacco products on periodontal and peri-implant health status. Indeed, a better comprehension of the role of these alternative smoking habits, which may affect periodontitis and peri-implantitis onset differently from traditional tobacco use, may pave the way for multi-disciplinary personalized prevention strategies, especially in subjects considered at higher risk, such as those who are diabetic [79,80,81,82]. Moreover, the indirect effect of both E-Cigs and HNB tobacco products on periodontitis and peri-implant treatment outcomes may encourage the use of adjunctive therapies, also comprising antibiotics and oral antiseptics administration in non-conventional smokers [80,83,84,85,86].

## 5. Conclusions

The presented results carefully support the hypothesis that e-cigarettes may cause attenuated clinical inflammatory signs of periodontitis, and, hypothetically, of peri-implantitis, when compared to conventional tobacco smoke. However, both electronic cigarettes and Heat-Not-Burn tobacco, considered as alternative smoking products, containing nicotine, may have negative effects on periodontal and peri-implant health, as demonstrated in vitro by the toxic effects at the cellular level detected.

Furthermore, a deeper insight into the existence and extent of the effect putatively exerted by E-Cigs and HNB tobacco products on periodontitis progression rate, as already estimated for traditional tobacco use, may guide in the optimal planning of active periodontal treatment sessions and, above all, of maintenance phase recall intervals.

## Figures and Tables

**Table 1:** Excluded studies and reason for exclusion.

Authors, Year	Reason for Exclusion
Yang, 2020 [23]	Systematic review
Ralho, 2019 [22]	Systematic review
Javed, 2017 [33]	No clinical/radiographic parameters
Shaito, 2017 [34]	No clinical/radiographic parameters
Vyncke, 2020 [35]	Systematic review
Karina, 2020 [36]	Review
Atuegwu, 2019 [37]	No clinical/radiographic parameters
Chrcanovic, 2015 [11]	Systematic review
Sancilio, 2015 [38]	No clinical/radiographic parameters
Ryder, 2018 [12]	Relationship between e-cigarettes and alcohol
Javed, 2019 [39]	No clinical/radiographic parameters
Ganesan, 2020 [40]	Relationship between e-cigarettes and oral microbiome
Andrikopoulos, 2019 [41]	No clinical/radiographic parameters
Nelson, 2019 [42]	Relationship between e-cigarettes and oral microbiome
Willershausen, 2014 [43]	No clinical/radiographic parameters
Rouabhia, 2018 [44]	No clinical/radiographic parameters
Holliday, 2019 [45]	Study on tobacco cessation and starting e-cigarettes
Zanetti, 2016 [46]	No clinical/radiographic parameters
Sandar, 2016 [27]	No clinical/radiographic parameters
Tatullo, 2016 [47]	Study on tobacco smokers which started to use E-cigarette
Alqahtani, 2020 [48]	No clinical/radiographic parameters
Al Rifaiy, 2018 [49]	Effect of the antimicrobial photodynamic therapy (aPDT) individuals vaping electronic cigarettes
Figueredo, 2021 [25]	Systematic review

**Table 2:** Characteristics of the studies included in the present systematic review: author(s) and year of publication; study design; total number, mean age, gender ratio and smoking habits of participants; periodontal status, number of implants, and periodontal and peri-implant clinical, radiographic and crevicular parameters, including clinical attachment loss (CAL) probing depth (PD), bleeding on probing (BoP), plaque index (PI), gingival index (GI), marginal bone levels (MBL), cytokines profile and periodontal treatment. Electronic devices intended for “vaping” were heterogeneously defined by the authors of the included studies and are currently named as “electronic cigarettes”.

Authors, Year Reference Study Design Aim/Objective	Population Sample Size Mean Age Gender Ratio	Periodontal and Peri-Implant Assessment Time Points Treatment Dental Implants (n.) Clinical Parameters Radiographic Parameters Crevicular Parameters Other Parameters	Main Results	Conclusions
Mokeem, 2018 [50] Case-control To compare PI, BOP, PD, CAL, MBL and cotinine, interleukin IL-1β and IL-6 levels among CS, WS, E-Cigs and NS	39 CS (42.4 ± 5.6 y.o.) 40 E-Cigs (44.7 ± 4.5 y.o.) 37 E-Cigs (28.3 ± 3.5 y.o.) 38NS (40.6 ± 4.5 y.o.) All males	Periodontal and peri-implant assessment at baseline No periodontal treatment No dental implants PI BoP CAL PD MBL IL-1b IL-6 cotinine	PI was significantly higher among CS and WS than E-Cigs (*p* < 0.05) and NS (*p* < 0.05).PI was significantly higher among E-Cigs than NS (*p* < 0.05). BOP were significantly higher among NS compared with CS (*p* < 0.05) and WS (*p* < 0.05) and E-Cigs (*p* < 0.05) PD (*p* < 0.05), CAL (*p* < 0.05) and MBL (*p* < 0.05) were significantly higher among CS and WS compared with E-Cigs and NS. There was no difference in PD, CAL, MBL, IL-1β and IL-6 levels among E-Cigs and NS IL-1β (*p* < 0.01) and IL-6 (*p* < 0.01) levels were significantly higher among CS, WS than E-Cigs and NS. Cotinine levels were significantly higher among CS (*p* < 0.001) and WS (*p* < 0.001) and E-Cigs (*p* < 0.001) than NS.	CS and WS had worse radiographic parameters of periodontal inflammation than E-Cigs and NS. Cotinine levels were similar in all groups. Salivary IL-1β and IL-6 levels were higher in CS and WS than E-Cigs and NS
Al-Aali, 2018 [51] Case-control To compare clinical and radiographic peri-implant parameters and TNF-a and IL-1b levels among E-Cigs and NS	47 E-Cigs (35.8 ± 6.2 y.o.) 45 NS (42.6 ± 2.7 y.o.) All males	Periodontal and peri-implant assessment at baseline No periodontal treatment Dental implants n. 125 (at least one positioned for ≥36 months) PI BoP PD (>/=4 mm) PIBL TNF-α IL-1β PISF volume	BOP was statistically significantly higher in NS compared to E-Cigs (*p* < 0.01). PD (*p* < 0.05); PIBL (*p* < 0.05);TNF-a (*p* < 0.001) and IL-1b (*p* < 0.01) were statistically significantly higher in E-Cigs than NS. There was a significant positive relationship among TNF-a levels and BOP (P5.024) and PIBL (P5.016); and among IL-1b and PIBL (P5.018) in E-Cigs.	Clinical and radiographic peri-implant parameters are worse among E-Cigs than NS. E-Cigs show higher levels of pro-inflammatory cytokines than NS.
Karaaslan, 2020 [52] Case-control To evaluate the effects of vaping, cigarettes smoke and smoking cessation on periodontal clinical parameters, oxidative stress markers and pro-inflammatory cytokines in patients with periodontal disease	19 CS (35.26 ± 2.31 y.o.) 19 E-Cigs (34.74 ± 2.38 y.o.) Ex- cigarettes smokers: 19 individuals (35.58 ± 2.04 y.o.) 39M/18F	Periodontal and peri-implant assessment at baseline No periodontal treatment No dental implants PI GI CAL IL-8 TNF-a GsH-Px (Glutathione peroxidase) 8-OHdG (8-hydroxydeoxyguanosine)	No significant differences were found between the groups for PD, PI, CAL. GI was significantly higher in group 2e 3 than group 1 and GI was significantly lower in group 2 than group 3. IL-8 level of Group I (70.47 ± 2.76) was significantly lower than in Groups II and III. TNF-a level of Group I (4.20 ± 0.14) was significantly higher than in Groups II and III.	Cigarette smoke and vaping have negative effects on the markers of oxidative stress and inflammatory cytokines.
AlQahtani, 2018 [53] Case-control To compare clinical and radiographic peri-implant parameters and cytokines among CS, WS, E-Cigs, and NS	40 CS 40 WS 40 E-Cigs 40 NS Mean age: 41.8 y.o. All males	Periodontal and peri-implant assessment at baseline No periodontal treatment Dental implants n. 253 (at least one in service for >/=36 months) PI BoP PD (≥4 mm) MBL TNF-a IL-6 IL-1B PISF	Peri-implant PI (*p* < 0.05), PD (*p* < 0.05) and RBL (*p* < 0.01) was significantly higher among CS, WS and E-Cigs compared to NS. BOP in CS, WS, and E-Cigs showed statistical differences (*p* < 0.01) compared to NS CS and WS showed significantly higher PD ≥ 4 mm and RBL compared with E-Cigs (*p* < 0.05). TNF-α, IL-6, and IL-1β were significantly higher in CS, WS, and E-Cigs than NS. No statistical differences for cytokines were observed among CS and WS.	Tobacco smoking is associated with poor peri-implant health.
BinShabaib, 2019 [54] Case-control To compare clinical periodontal status and gingival crevicular fluid cytokine profile among CS, E-Cigs and NS	46 CS (29.5 ± 5.8 y.o.) 43M:3F 44 E-Cigs (27.8 ± 3.1 y.o.) 42M:2F 45NS (30.2 ± 4.4 y.o.) 39M:6F	Periodontal and peri-implant assessment at baseline No periodontal treatment No dental implants PI BoP PD CAL MT MBL IL-1b IL-6 TNF-a MMP-8 IFN-g	PI (*p* < 0.05), PD (*p* < 0.05) and clinical AL (*p* < 0.05) were significantly higher among individuals in CS than NS. BOP was manifested more among NS than in CS (*p* < 0.05) and E-Cigs (*p* < 0.05). MBL was significantly higher in CS (*p* < 0.01) and E-Cigs (*p* < 0.01) than NS The concentrations of IL-1β, IL-6, IFN-γ, TNF-α and MMP-8 were significantly higher in the GCF samples of CS (*p* < 0.05) than E-Cigs and NS.	Periodontal status is worse and GCF levels of pro-inflammatory cytokines are higher in CS compared with E-Cigs and NS.
Vohra, 2020 [55] Case-control To compare self-rated oral symptoms and clinical and radiographic periodontal status among CS, E-Cigs, and NS	28 CS (33.3 ± 2.2 y.o.) 26 E-Cigs (31.8 ± 2.05 y.o.) 26 NS (33.5 ± 1.4 y.o.) All males	Periodontal and peri-implant assessment at baseline No periodontal treatment No dental implants PI BoP CAL PD (>/=4 mm) MT MBL	PI (*p* < 0.05) and PD (*p* < 0.05) were increased in CS than E-Cigs and NS. There was no statistically significant difference in BOP, CAL, MBL and MT among the four groups.	PI and PD are increased in CS than other groups. Pain in teeth and gums are more often perceived by CS than other groups.
Javed, 2017 [56] Cohort To compare clinical periodontal parameters among CS, E-Cigs and NS	33 CS (41.3 ± 2.8 y.o.) 31 E-Cigs (37.6 ± 2.1 y.o.) 30 NS (40.7 ± 1.6 y.o.) All males	Periodontal and peri-implant assessment at baseline No periodontal treatment No dental implants PI BoP CAL PD (>/=4 mm) MT MBL	PI (*p* < 0.01), and PD ≥ 4 mm (*p* < 0.01) were significantly higher in CS and in E-Cigs compared to NS. BOP was significantly higher in NS than CS (*p* < 0.01) and E-Cigs (*p* < 0.01). There was no difference in MT, CAL and MBL among the groups.	CS show worse clinical periodontal parameters compared with E-Cigs and NS.
Jeong, 2020 [26] Case-control To evaluate the association of CS and E-Cigs with periodontal disease	Total number: 13,551 With and without periodontal disease CS E-Cigs Ex-CS NS ≥18 y.o. 5715M/7836F	Periodontal and peri-implant assessment at baseline (data between 2013 and 2015) No periodontal treatment No dental implants CPI	Periodontal disease was more prevalent in E-Cigs and CS than NS. E-Cigs and CS had higher dental disease (dental caries, toothache and dental damages) than NS.	E-Cigs and CS were significantly associated with increased periodontal disease. So, vaping may not be a healthy alternative to cigarette smoke.
Aldakheel, 2020 [57] Case-control To compare pathogenic bacteria, count from the subgingival biofilm of CS and E-Cigs with periodontitis with that of NS with and without periodontitis	15 CS (40.5 ± 2.1 y.o.) 5 E-Cigs (38.6 ± 3.3 y.o.) 15NS with periodontitis (39.4 ± 1.6 y.o.) 15 NS without periodontitis (39.5 ± 0.8 y.o.) M/F: MD	Periodontal and peri-implant assessment: MD No periodontal treatment No dental implants PI GI PD CAL MBL	The scores of PI (*p* < 0.001), GI (*p* < 0.001), CAL (*p* < 0.001), PD (*p* < 0.001) and mesial (*p* < 0.001) and distal (*p* < 0.001) MBL were significantly higher among CS, E-Cigs, and NS with periodontitis compared with NS without periodontitis. There was no statistically significant difference in GI, PD, CAL, mesial and distal MBL and PI among CS, E-Cigs, and NS with periodontitis.	E-Cigs and CS have the same number of pathogenic bacteria in the oral-biofilm.
Ibraheem, 2020 [58] Case-control To compare the levels of Receptor activator of NF-kappa B ligand (RANKL) and osteoprotegerin in the gingival crevicular fluid of CS, WS, E-Cigs, NS	30 CS (46.5 ±5.3 y.o.) 30 WS (45.5 ±4.4 y.o.) 30 E-Cigs (45.6 ± 3.6 y.o.) 30 NS (3.8 + 1.7 y.o.) All males	Periodontal and peri-implant assessment: MD No periodontal treatment No dental implants PI BoP PD CAL MBL RANKL OPG	PI (*p* < 0.01) and PD (*p* < 0.01) were significantly higher among CS, WS, E-Cigs than NS. The GCF volume was significantly higher among CS (0.92 ± 0.05 μL) (*p* < 0.01) and WS (0.66 ± 0.08 μL) (*p* < 0.001) and E-Cigs (0.62 ± 0.03 μL) (*p* < 0.01) than NS (0.21 ± 0.007 μL). The RANKL levels were significantly higher among CS (14.9 ± 8.2 pg/mL) (*p* < 0.001) and WS (12.6 ± 8.8 pg/mL) (*p* < 0.01) and E-Cigs (11.5 ± 8.4 pg/mL) (*p* < 0.01) than NS (3.5 ± 0.7 pg/mL). The OPG levels were significantly higher among CS (95.9 ± 7.2 pg/mL) (*p* < 0.001) and WS (86.6 ± 5.8 pg/mL) (*p* < 0.01) and E-Cigs (77.5 ± 3.4 pg/mL) (*p* < 0.05) than NS (21.5 ± 10.7 pg/mL). There was no significant difference in RANKL and OPG levels among CS, WS and E-Cigs.	CS, WS and E-Cigs have higher levels of RANKL and OPG in the GCF than NS.
ALHarthi, 2019 [59] Prospective To investigate the impact of cigarette smoking and vaping on periodontal tissues after a full-mouth ultrasonic scaling	30 CS (36.4 ± 2.8 y.o.) 28 E-Cigs (32.5 ± 4.8 y.o.) 31 NS (32.6 ± 3.5 y.o.) All males	Periodontal and peri-implant assessment at baseline and after 3 and 6 months of follow-up Scaling No dental implants PI BoP PD >/= 4 mm CAL MT	At baseline, BOP was significantly higher in NS than CS and E-Cigs (*p* < 0.01). In CS, there was no statistically significant difference in mean PI and PD at 6 months’ follow-up compared with baseline and 3 months follow-up. In E-Cigs and NS, there was no significant difference in PI, BOP, and PD at 3 months’ (*p* > 0.05) and 6 months’ (*p* > 0.05) follow-up.	GI is worse in CS compared with E-Cigs and NS after FMUS
ArRejaie, 2018 [60] Case-control To compare clinical and radiographic peri-implant parameters and levels of MMP-9 and IL-1b among CS, E-Cigs and NS	32 CS (40.4 ± 3.5) 31 E-Cigs (35.8 ± 6.2 y.o.) 32 NS (42.6 ± 2.7 y.o.) All males	Periodontal and peri-implant assessment at baseline No periodontal treatment Dental implants n. 159 (at least one positioned for >= 36 months) PI BoP PD >/= 4 mm MBL IL-1b MMP-9	BOP was significantly higher in NS compared with CS and E-Cigs (*p* < 0.01). PI (*p* < 0.01), PD (*p* < 0.01),MMP-9 (*p* < 0.001) and IL-1b levels (*p* < 0.01) were significantly higher in CS and E-Cigs than NS. MBL was significantly higher in CS compared with E-Cigs and NS (*p* < 0.01). Significant positive associations were found between MMP-9 (*p* = 0.0198) and IL-1b (*p* = 0.0047) levels and MBL in CS; and a significant positive association between IL-1b and MBL in E-Cigs (*p* = 0.0031).	Higher levels of cytokines in CS and E-Cigs suggest greater peri-implant inflammatory response and so worse clinical and radiographic peri-implant parameters.
Al-Hamoudi, 2020 [61] Cross-sectional To investigate the effect of scaling and root planing on inflammatory cytokines IL-4, IL-9, IL-10, and IL-13 in E-Cigs and NS with periodontitis	36 E-Cigs (47.7± 5.8 y.o.) 35NS (46.5 ± 3.4 y.o.) 62M/9F	Periodontal and peri-implant assessment at baseline and after 3 months of follow-up Scaling and root planing No dental implants PI GI PD CAL MBL IL-4 IL-9 IL-10 IL-13 Crevicular fluid volume	At baseline, there were no differences in PI, PD, CAL, MBL, and GCF IL-4, IL-9, IL-10, and IL-13 among E-Cigs and NS. At the 3-month follow-up, there were no significant differences in PI, GI, PD, CAL and MBL in E-Cigs compared to baseline, while there were significant reductions in PI, GI, and PD among NS. At the 3-month follow-up, GCF IL-4, IL-9, IL-10, and IL-13 levels were significantly elevated in E-Cigs and in NS (*p* < 0.05) compared to baseline.After3-month, GCF IL-4, IL-9, IL-10, and IL-13 levels were significantly higher in NS (*p* < 0.05) than in E-Cigs	Levels of GCF IL-4, IL-9, IL-10, and IL-13 increased after SRP in E-Cigs and NS with CP.
Alqahtani, 2019 [62] Cross-sectional To compare cotinine levels in the PISF among CS, WS, E-Cigs and NS	35 CS (36.3 ± 1.2 y.o.) 33 WS (34.1 ± 1.4 y.o.) 34 E-Cigs (33.5 ± 0.7 y.o.) 35 NS (32.2 ± 0.6 y.o.) All males	Periodontal and peri-implant assessment at baseline No periodontal treatment Dental implants n.137 PI PD BoP Cotinine in the PISF	PI (*p* < 0.05) and PD (*p* < 0.05) were significantly higher in CS, WS and E-Cigs than NS BoP was higher in NS compared with CS (*p* < 0.05), WS (*p* < 0.05) and E-Cigs (*p* < 0.05). PISF and cotinine levels were significantly higher among CS (*p* < 0.05) and WS (*p* < 0.05) and E-Cigs (*p* < 0.05) than NS.	Nicotine increases the expression of cotinine in the PISF.
Sinha, 2020 [63] Case-control To evaluate PI, BoP, PD, TNF-α and IL-1b levels among E-Cigs and NS	47 E-Cigs (34.6 ± 6.1 y.o.) 45 NS (44.8 ± 2.5 y.o.) All males	Periodontal and peri-implant assessment at baseline No periodontal treatment Dental implants n. 66 for E-Cigs and 55 for NS PI BoP PD PIBL IL1b TNF-a PISF volume	BOP was significantly higher in NS than E-Cigs. PD and PIBL were significantly higher in E-Cigs than NS. TNF- α levels and IL-1β levels were significantly higher in E-Cigs than NS PISF concentrations were also found relatively higher in E-Cigs than NS.	E-Cigs show PD, PIBL and TNF-α levels and IL-1β levels worse than NS
Al Deeb, 2020 [64] Case-control To assess the effectiveness of Photodynamic therapy in the treatment of peri-implant mucositis in CS, E-Cigs and NS	25 CS (29.5 ± 5.8 y.o.) 21 E-Cigs (27.8 ± 3.1 y.o.) 25 NS (0.2 ± 4.4 y.o.) All males	Periodontal and peri-implant assessment at baseline and after 12 weeks Photodynamic therapy at baseline + mechanical debridement (MD) Dental implants n. 111 implants (at least one in service for ≥1.5 years) PI BoP PD TNF-a MMP-8 PISF volume	PI, PD, MMP-8 and TNF-a were higher in CS and E-Cigs than NS at baseline. BOP was higher in NS than other groups. A statistically significant reduction in PI and PD parameters was observed on baseline and at 12 weeks in all groups. BOP significantly increased in group 1 and 2 at 12 weeks. A statistically significant reduction from baseline to 12 weeks was reported in the biomarker levels for all the study groups.	PDT with adjunctive mechanical debridement reduced PI and PD and cytokines but increased BPO
AlJasser, 2021 [65] Case-control To evaluate the adverse effects of E-Cigs on periodontal health	30 CS (46.9 M, 46.6 F y.o.) 32 E-Cigs (36 M, 46.8 F y.o.) 38 NS (28.6 M, 46.9 F y.o.) 70M/30F	Periodontal and peri-implant assessment at baseline, after1 month, 6 months and 1 year Surgical periodontal treatment No dental implants PD BOP CAL Cotinine levels saliva flow CO (carbon monoxide)	BOP and PD increased in all three groups, but CAL uniquely increased in E-Cigs. CS have higher carbon monoxide and salivary cotinine levels than other groups.	Among the recruited participants, CAL after 6 months was significantly worse only in the E-Cigs
Fangxi Xu, 2021 [66] Case-control To compare periodontal parameters among CS, E-Cigs and NS after peri-implantitis treatment	20 CS (54.1 y.o.) 20 E-Cigs (46.8 y.o.) 20 NS (46.9 y.o.) 31M/29F	Periodontal and peri-implant assessment at baseline and after 6 months No periodontal treatment Dental implants n. 60 PD BOP PI IL-1b IL-6 MMP-8 TIMP-1	The PI of 100% of NS changed to ‘0′ and 35% change in cigarettes and 30% change in E-Cigs which is statistically significant (*p* = 0.016). The mean values of PD have shown statistically significant change across the three groups over the four time intervals of observation. The comparison of mean values of IL-1 β, IL-6 and TIMP-1 showed statistically significant change across the three groups over the four intervals of observation (*p* < 0.0001).	Vaping was found to be the most prevalent risk indicator for peri-implantitis.

Abbreviations: traditional tobacco or cigarette smokers, CS; electronic cigarette or electronic cigarette smokers, E-Cigs; non-smokers, NS; water piper smokers, WP; Years old, y.o.; Missing data, MD; Plaque index, PI; Bleeding on Probing, BOP; Probing Depth, PD; Clinical Attachment Loss, CAL; MT, number of missing teeth; Marginal Bone Loss (MBL); Community periodontal index, CPI; Peri-implant bone loss, PIBL; Peri-implant sulcular fluid, PISF; Interleukin, IL; Interferon-gamma, INF-g; Tumor Necrosis Factor-a, TNF-a; Tissue inhibitor metalloproteinase-1, TIMP-1.

**Table 3:** Reported results on clinical and radiographic periodontal and peri-implant parameters.

Periodontal Clinical and Radiographic Parameter	Author, Year Reference Study Design	Main Result(s)	Considerations
CAL	Mokeem, 2018 [50] Case-control	CAL (*p* < 0.05) was significantly higher among CS and WS compared to E-Cigs and NS. There was no difference in CAL, between E-Cigs and NS	CS and WS have worse CAL values compared to E-Cigs and NS; E-Cigs and NS have no significant difference in CAL values
CAL	BinShabaib, 2019 [54] Case-control	CAL (*p* < 0.05) was significantly higher among CS than NS. No differences were among E-Cigs and NS	Cigarette smoke negatively affects the CAL values
CAL	Vohra, 2020 [55] Case-control	There was no statistically significant difference in CAL, among the CS, E-Cigs and NS	CS, E-Cigs and NS have similar CAL values
CAL	Aldakheel, 2020 [57] Case-control	The scores of CAL (*p* < 0.001) was significantly higher among CS, E-Cigs, and NS with periodontitis compared with NS without periodontitis. There was no statistically significant difference in CAL among CS, E-Cigs, and NS with periodontitis	Periodontal bacteria negatively influence CAL values
CAL	Al-Hamoudi, 2020 [61] Cross-sectional	At baseline and after 3 months of follow up there were no significant differences in CAL between the E-Cigs and NS	E-Cigs do not have differences with NS for the CAL
CAL	ALHarthi, 2018 [59] Prospective	The levels of CAL remained unchanged in all groups	CAL remains unchanged among the groups
CAL	Javed, 2017 [56] Cohort	There was no statistically significant difference in CAL among individuals among the groups	Smoke does not negatively influence CAL
CAL	Karaaslan, 2020 [52] Case-control	There were No significant differences among the groups for mean AL	Smoke does not negatively influence CAL
CAL	Ibraheem, 2020 [58] Case-control	CAL was significantly higher in CS, WS and E-Cigs compared with NS	Smoke negatively affects CAL values
CAL	Fangxi Xu, 2021 [66] Case-control	CAL increased in E-Cigs	Vaping negatively affects CAL values
BoP	Mokeem, 2018 [50] Case-control	Percentage of sites with BOP were significantly higher among NS compared with CS and WS and E-Cigs. There was no statistically significant difference in BOP among CS and WS and E-Cigs	Smoke improves BoP
BoP	Vohra,2020 [55] Case-control	There was no statistically significant difference in BOP, among the four groups	Smoke does not influence BoP
BoP	Javed, 2017 [56] Cohort	BOP was significantly higher in NS than CS (*p* < 0.01) and E-Cigs (*p* < 0.01)	Smoke improves BoP
BoP	Ibraheem, 2020 [58] Case-control	There was no statistically significant difference in BOP among individuals in all groups	Smoke does not improve BoP
BoP	ALHarth, 2018 [59] Prospective	At baseline, BOP was significantly higher in NS than CS and E-Cigs (*p* < 0.01). In E-Cigs and NS, there was no significant difference in BOP at 3 months’ (*p* > 0.05) and 6 months’ (*p* > 0.05) follow-up	Cigarette smoke improves Bop
Bop	Fangxi Xu, 2021 [66] Case-control	BoP similarly increased over time in all three groups	BoP changes in all groups
BoP	BinShabaib, 2019 [54] Case-control	BOP was manifested more among NS than in CS (*p* < 0.05) and E-Cigs (*p* < 0.05). No differences were among E-Cigs and NS	BoP was more often manifested among never smokers
PI	Mokeem, 2018 [50] Case-control	Percentage of sites with plaque were significantly higher among CS and WS compared with E-Cigs (*p* < 0.05) and NS (*p* < 0.05). Percentage of sites with plaque were significantly higher among E-Cigs compared to NS (*p* < 0.05). There was no statistically significant difference in PI among CS and WS and E-Cigs	Smoke increases plaque accumulation.
PI	Ibraheem, 2020 [58] Case-control	PI (*p* < 0.01) was significantly higher among CS, WS and E-Cigs than NS	Smoke gets worse PI
PI	Aldakheel, 2020 [57] Case-control	The scores of PI (*p* < 0.001) were significantly higher between CS, E-Cigs, and NS with periodontitis compared with NS without periodontitis. There was no statistically significant difference in PI among CS, E-Cigs, and NS with periodontitis	Smoke gets worse periodontitis
PI	ALHarthi, 2018 [59] Prospective	In CS, there was no statistically significant difference in mean PI at 6 months’ follow-up compared with baseline and 3 months’ follow-up. In E-Cigs and NS, there was no significant difference in PI at 3 months’ (*p* > 0.05) and 6 months’ (*p* > 0.05) follow-up	Vaping does not influence PI
PI	BinShabaib, 2019 [54] Case-control	PI (*p* < 0.05), was significantly higher among CS than NS. No differences were among E-Cigs and NS	Smoke gets worse plaque index
PI	Al-Hamoudi, 2020 [61] Cross-sectional	At baseline, there were no differences in PI, among E-Cigs and NS. At the 3-month follow-up, there were no significant differences in PI, in ES compared to baseline, while there were significant reductions in PI, among NS	PI is better among NS after 3-month follow-up
PI	Vohra, 2020 [55] Case-control	PD (*p* < 0.05) was increased in CS than E-Cigs and NS	Cigarettes smoke gets worse plaque index
PI	Karaaslan, 2020 [52] Case-control	No significative differences were found between the groups for PI	Smoke does not influence PI
PD	Vohra, 2020 [55] Case-control	PD (*p* < 0.05) was increased in CS than E-Cigs and NS.	Cigarette smoke gets worse PD
PD	Javed, 2017 [56] Cohort	PD ≥ 4 mm (*p* < 0.01) were significantly higher in CS and in E-Cigs compared with NS	Smoke influences PD
PD	Ibraheem, [58] Case-control 2020	PD (*p* < 0.01) was significantly higher among CS, WS, and E-Cigs than NS	Smoke gets worse PD
PD	Aldakheel, 2020 [57] Case-control	The scores of PD (*p* < 0.001) were significantly higher among CS, E-Cigs, and NS with periodontitis compared with NS without periodontitis. There was no statistically significant difference in, PD, among CS, E-Cigs, and NS with periodontitis.	Smoke gets worse periodontitis
PD	ALHarthi, 2018 [59] Prospective	In CS, there was no statistically significant difference in PD at 6 months’ follow-up compared with baseline and 3 months’ follow-up. In E-Cigs and NS, there was no significant difference in PD at 3 months’ and 6 months’ follow-up	Vaping does not influence PD
PD	BinShabaib, 2019 [54] Case-control	PD (*p* < 0.05) was significantly higher among individuals in CS than NS. No differences were among E-Cigs and NS	Smoke gets worse PD
PD	Al-amoudi, 2020 [61] Cross-sectional	At baseline, there were no differences in PD, among E-Cigs and NS. At the 3-month follow-up, there were no significant differences in PD in E-Cigs compared to baseline values, but there was a statistically significant reductions in PD among NS	SRP improves PD in NS
PD	Mokeem, 2018 [50] Case-control	There was no statistically significant difference in PD among E-Cigs c and NS PD was significantly higher among CS and WS compared with E-Cigs and NS.	Vaping does not influence PD
PD	Karaaslan, 2020 [52] Case-control	No significative differences were found between the groups for PD	Smoke does not influence PD
PD	Fangxi Xu, 2021 Case-control	PD similarly increased over time in all three groups	PD increased in all three groups
GI	Al-amoudii, 2020 [61] Cross-sectional	At baseline, GI was significantly higher in NS than E-Cigs; at the 3-month follow-up, there were no significant differences in GI, in E-Cigs compared to baseline, while there were statistically significant reductions in GI among NS.	Vaping improves gingival conditions
GI	Karaaslan, 2020 [52] Case-control	GI was significantly higher in E-Cigs and EX-CS than CS and GI was significantly lower in group E-Cigs than EX-CS	Smoke improves GI
GI	Aldakheel, 2020 [57] Case-control	GI (*p* < 0.001) was significantly higher among CS, E-Cigs, and NS with periodontitis compared with NS without periodontitis. There was no statistically significant difference in GI, among CS, E-Cigs, and NS with periodontitis	GI is worst among subjects with periodontitis
MBL	Vohra, 2020 [55] Case-control	There was no statistically significant difference in MBL among CS, E-Cigs and NS	Smoke does not influence MBL among the groups
MBL	Ibraheem, 2020 [58] Case-control	MBL (*p* < 0.01) was significantly higher among CS, WS, E-Cigs than NS	Smoke gets worse bone loss
MBL	Aldakheel, 2020 [57] Case-control	The scores mesial (*p* < 0.001) and distal (*p* < 0.001) MBL were significantly higher among CS, E-Cigs, and NS with periodontitis compared with NS without periodontitis. There was no statistically significant difference in mesial and distal MBL among CS, E-Cigs, and NS with periodontitis	Smoke gets worse periodontitis
MBL	BinShabaib, 2019 [54] Case-control	MBL was significantly higher in CS (*p* < 0.01) and E-Cigs (*p* < 0.01) than NS No differences were among E-Cigs and NS	Smoke gets worse bone loss
MBL	Al-Hamoudi, 2020 [61] Cross-sectional	At baseline, there were no differences in MBL among E-Cigs and NS. At the 3-month follow-up, there were no statistically significant differences in MBL between the two groups	Vaping does not influence MBL
MBL	Mokeem, 2018 [50] Case-control	MBL were significantly higher among CS and WS compared to E-Cigs and NS There was no difference in MBL among E-Cigs and NS	Smoke gets worse bone loss
MBL	Javed, 2017 [56] Cohort	There was no difference in MBL among the groups	No differences among the groups
MT	Javed, 2017 [56] Cohort	There was no difference in MT among the groups	Smoke does not influence MT
MT	Vohra, 2020 [55] Case-control	There was no statistically significant difference in MT among CS, E-Cigs, NS	Smoke does not influence MT
MT	ALHarthi, 2018 [59] Prospective	There was no difference in the numbers of MT in all groups	Smoke does not influence MT
MT	BinShabaib, 2019 [54] Case-control	No differences among the groups	Smoke does not influence MT

**Table 4:** Reported results on crevicular inflammatory periodontal parameters.

Periodontal Inflammatory Parameter	Author, Year Reference Study Design	Main Result(s)	Considerations
IL-1b	Mokeem, 2018 [50] Case-control	There was no difference in IL-1β levels among E-Cigs and NS IL-1β (*p* < 0.01) levels were significantly higher among CS, WS than E-Cigs and NS	Cigarette and pipe smoke increase gingival inflammation
IL-1b	BinShabaib, 2019 [54] Case-control	The concentration of IL-1β was significantly higher in the GCF samples of CS (*p* < 0.05) than E-Cigs and NS. No differences were among E-Cigs and NS	Cigarettes smoke increase inflammation
IL-6	Bin Shabaib, 2019 [54] Case-control	The concentration of IL-6 was significantly higher in the GCF samples of CS (*p* < 0.05) than E-Cigs and NS. No differences were among E-Cigs and NS	Cigarettes smoke increase inflammation
IL-6	Mokeem, 2018 [50] Case-control	IL-6 (*p* < 0.01) levels were significantly higher among CS, WS than E-Cigs and NS	Cigarette smoke increases gingival inflammation
TNF-a	Bin Shabaib,2019 [54]	The concentration of TNF-α was significantly higher in the GCF samples of CS (*p* < 0.05) than E-Cigs and NS. No differences were among E-Cigs and NS	Cigarettes smoke increase inflammation
TNF-a	Karaaslan, 2020 [52] Case-control	TNF-a level of Group CS (4.20 +/− 0.14) was significantly higher than E-Cigs	Smoke increases gingival inflammation
COTININE	Mokeem, 2018 [50] Case-control	Cotinine levels were significantly higher among CS (*p* < 0.001) and WS (*p* < 0.001) and E-Cigs (*p* < 0.001) than NS	Smoke increases gingival inflammation
COTININE	Fangxi Xu, 2021 [66] Case-control	Salivary cotinine levels was highest among CS	CS have highest salivary cotinine levels
IL-8	Karaslaan, 2020 [52] Case-control	IL-8 level of CS (70.47 +/− 2.76) was significantly lower than in E-Cigs and FS	Smoke improves il-8 levels
MMP-8	BinShabaib, 2019 [54] Case-control	The concentrations of MMP-8 were significantly higher in the GCF samples of CS (*p* < 0.05) than E-Cigs and NS. No differences were among E-Cigs and NS	Cigarettes smoke increase inflammation
IFN-y	BinShabaib, 2019 [54] Case-control	The concentration of I FN-γ was significantly higher in the GCF samples of CS (*p* < 0.05) than ES and NS. No differences were among E-Cigs and NS	Cigarettes smoke increase inflammation
CPI	Jeong, 2020 [26]	Periodontal disease was more prevalent in E-Cigs and CS than NS	E-Cigs and CS were each significantly associated with increased periodontal disease rates. This study suggests that vaping may not be a safe alternative to smoking
IL-4	Al-Hamoudi, 2020 [61] Cross-sectional	At baseline, there were no differences, and GCF IL-4 among E-Cigs and NS. At the 3-month follow-up, GCF IL-4 levels were significantly elevated in ES and in NS (*p* < 0.05) compared to baseline. After3-month, GCF IL-4, levels were significantly higher in NS (*p* < 0.05) than in E-Cigs	Levels of GCF IL-4 increased after SRP in E-Cigs and NS with CP; however, the anti-inflammatory effect of SRP was more profound in NS than in E-Cigs
IL-9	Al-Hamoudi, 2020 [61] Cross-sectional	At baseline, there were no differences in IL-9, among E-Cigs and NS. At the 3-month follow-up, GCF IL-9 levels were significantly elevated in E-Cigs and in NS (*p* < 0.05) compared to baseline. After 3 months, GCF IL-9 levels were significantly higher in NS (*p* < 0.05) than in E-Cigs	Levels of GCF IL-9 increased after SRP in E-Cigs and NS with CP; however, the anti-inflammatory effect of SRP was more profound in NS than in E-Cigs
IL-10	Al-Hamoudi, 2020 [61] Cross-sectional	At baseline, there were no differences in IL-10 among E-Cigs and NS. At the 3-month follow-up, GCFIL-10 levels were significantly elevated in E-Cigs and in NS (*p* < 0.05) compared to baseline.After3-month, GCF IL-10, was significantly higher in NS (*p* < 0.05) than in E-Cigs	Levels of GCF IL-10 increased after SRP in E-Cigs and NS with CP; however, the anti-inflammatory effect of SRP was more profound in NS than in E-Cigs
IL-13	Al-Hamoudi, 2020 [61] Cross-sectional	At baseline, there were no differences in IL-13 among E-Cigs and NS. At the 3-month follow-up, GCF IL-13 levels were significantly elevated in E-Cigs and in NS (*p* < 0.05) compared to baseline.After3-month, GCF IL-13 levels were significantly higher in NS (*p* < 0.05) than in E-Cigs	Levels of GCF IL-13 increased after SRP in E-Cigs and NS with CP; however, the anti-inflammatory effect of SRP was more profound in NS than in E-Cigs
CO	Fangxi Xu, 2021 [66] Case-control	CO levels was highest among CS	CS have highest CO levels
GSH-PX AND 8-OHdG	Karaaslan, 2020 [52] Case-control	Although the GSH-Px level of Group II was higher than Group I, this difference was not statistically significant, but the mean GSH-Px level of Group III was significantly higher than in Groups I and II. There was no significant association among the groups	CS and E-Cigs had the same unfavorable effects on the markers of oxidative stress and inflammatory cytokines
RANKL	Ibraheem, 2020 [58] Case-control	The RANKL levels were significantly higher among CS (14.9 ± 8.2 pg/mL) (*p* < 0.001) and WS (12.6 ± 8.8 pg/mL) (*p* < 0.01) and E-Cigs (11.5 ± 8.4 pg/mL) (*p* < 0.01) than NS (3.5 ± 0.7 pg/mL). There was no significant difference in RANKL among CS, WS and ES	CS and WS and E-Cigs are associated with an increased expression of RANKL in the GCF
OPG	Ibraheem, 2020 [58] Case-control	The OPG levels were significantly higher among CS (95.9 ± 7.2 pg/mL) (*p* < 0.001) and WS (86.6 ± 5.8 pg/mL) (*p* < 0.01) and E-Cigs (77.5 ± 3.4 pg/mL) (*p* < 0.05) than NS (21.5 ± 10.7 pg/mL)	CS and WS and E-Cigs are associated with an increased expression of OPG in the GCF

**Table 5:** Reported results on clinical and radiographic peri-implant parameters.

Clinical and Radiographic Peri-Implant Parameters	Author, Year Reference Study Design	Main Result(s)	Considerations
BoP	ArRejaie, 2018 [60] Case-control	Peri-implant BOP was significantly higher in NS compared with CS and E-Cigs (*p* < 0.01)	Smoke improves Bop
BoP	Al-Aali, 2018 [51] Case-control	BOP was statistically significantly higher in NS compared to E-Cigs (*p* < 0.01)	Vaping smoke improves Bop
BoP	Sinha, 2020 [63] Case-control	BOP was significantly higher in NS than E-Cigs	Vaping smoke improves Bop
BoP	Alqahtani, 2019 [62] Cross-sectional	BoP was higher in NS compared with CS (*p* < 0.05),WS (*p* < 0.05) and E-Cigs (*p* < 0.05)	Smoke improves Bop
BoP	Al Deeb, 2020 [64] Case-control	BOP was statistically significantly higher in NS than other groups	Smoke improves Bop
BoP	Alqahtani, 2019 [62] Cross-sectional	BOP in CS, WS, and E-Cigs showed statistical differences (*p* < 0.01) respect to NS	Smoke improves Bop
BoP	Al-Aali, 2018 [51] Case-control	BOP was statistically significantly higher in NS compared to E-Cigs (*p* < 0.01)	Vaping smoke improves Bop
BoP	AlJasser, 2021 [65]	The prevalence of BOP was observed in the three groups as 72%, (CS) 76.5% (E-Cigs) and 88.9% (NS) at baseline	Smoke improves Bop
PI	Alqahtani, 2019 [62] Cross-sectional	PI was significantly higher among individuals CS (*p* < 0.05), WS (*p* < 0.05), and E-Cigs (*p* < 0.05) compared with NS	Smoke gets worse plaque index
PI	ArRejaie, 2018 [60] Case-control	PI (*p* < 0.01 was significantly higher in CS and E-Cigs than NS. There were differences statistically significantly among CS and E-Cigs	Cigarettes smoke gets worse plaque index
PI	Al-Aali, 2018 [51] Case-control	PI showed no significant difference between NS and E-Cigs	Vaping does not influence PI
PI	Sinha, 2020 [63] Case-control	PI showed no significant difference between NS and E-Cigs	Vaping does not influence PI
PI	Alqahtani, 2019 [62] Cross-sectional	PI (*p* < 0.05) was significantly higher in CS, WS and E-Cigs than NS	Smoke gets worse plaque index
PI	Al Deeb, 2020 [64] Case-control	PI was higher in CS and E-Cigs than NS at baseline. Statistically significant reduction in PI was observed on baseline and at 12 weeks in all groups	Smoke gets worse plaque index
PI	AlJasser, 2021 [65] Case-control	PI of 100% of NS had changed to ‘0′ and 35% change in cigarettes and 30% change in E-Cigs which is statistically significant (*P* = 0.016)	PI was higher in NS than other groups
PD	ArRejaie, 2018 [60] Case-control	PD was significantly higher in CS and E-Cigs than NS	Smoke gets worse PD
PD	Al-Aali, 2018 [51] Case-control	PD was statistically significantly higher in E-Cigs than NS	Vaping gets worse PD
PD	Sinha, 2020 [63] Case-control	PD was significantly higher in E-Cigs than NS	Vaping gets worse PD
PD	Alqahtani, 2019 [62] Cross-sectional	PD was significantly higher among CS, WS and E-Cigs compared with NS. Among smokers, CS and WS showed significantly higher PD compared with E-Cigs	Smoke gets worse PD
PD	Alqahtani, 2019 [62] Cross-sectional	PD (*p* < 0.05) was significantly higher in CS, WS and E-Cigs than NS	Smoke gets worse PD
PD	Al Deeb, 2020 [64] Case-control	PD was higher in CS and E-Cigs than NS at baseline. Statistically significant reduction in PD parameter was observed on baseline and at 12 weeks in all groups	Smoke gets worse PD
PD	AlJasser, 2021 [65] Case-control	PD have shown statistically significant change across the three groups over the four-time intervals of observation (*P* = 0.024)	Smoke gets worse PD
MBL	Al-Aali, 2018 [51] Case-control	Peri-implant bone loss was statistically significantly higher in E-Cigs than NS	Smoke increases periodontal inflammation
MBL	ArRejaie, 2018 [60] Case-control	MBL was significantly higher in CS compared with E-Cigs and NS (*p* < 0.01). There were differences statistically significantly among CS and E-Cigs	Cigarettes smoke gets worse bone loss
MBL	Sinha, 2020 [63] Case-control	MBL was significantly higher in E-Cigs than NS	smoke gets worse bone loss
MBL	Alqahtani, 2019 [62] Cross-sectional	MBL (*p* < 0.01) was significantly higher among CS, WS and E-Cigs compared with NS. CS and WS showed significantly higher Peri-implant BL compared with E-Cigs (*p* < 0.05)	Cigarettes smoke gets worse bone loss
PD	ArRejaie, 2018 [60] Case-control	PD was significantly higher in CS and E-Cigs than NS	Smoke gets worse PD

**Table 6:** Reported results on crevicular inflammatory peri-implant parameters.

Inflammatory Periodontal Parameter	Author, Year Reference Study Design	Main Result(s)	Considerations
IL-1 b	ArRejaie, 2018 [60] Case-control	IL-1 b levels were statistically significantly higher in CS than E-Cigs and NS IL-1-b levels were statistically significantly higher in E-Cigs than NS	Smoke increase inflammation
IL-1 b	Alqahtani, 2019 [62] Cross-sectional	IL-1β was significantly higher in CS, WS, and E-Cigs than NS. No statistical differences for cytokines were observed between CS and WS	Smoke increases gingival inflammation
IL-1 b	Al-Aali, 2018 [51] Case-control	IL-1b (*p* < 0.01) was statistically significantly higher in E-Cigs than NS	Smoke increases gingival inflammation
IL-1 b	Sinha, 2020 [63] Case-control	IL-1 b levels was significantly higher in E-Cigs than NS	Smoke increases gingival inflammation
IL-1 b	AlJasser, 2021 [65] Case-control	Comparison of mean IL-1β values showed statistically significant variation between the three groups in the four observation intervals (*p* < 0.0001)	Smoke increases inflammation
IL-6	AlJasser, 2021 [65] Case-control	Comparison of mean IL-6 values showed a statistically significant change between the three groups in the four observation intervals (*p* < 0.0001)	Smoke increases inflammation
TNF-a	Sinha, 2020 [63] Case-control	TNF- α levels was significantly higher in E-Cigs than NS	Smoke increases inflammation
TNF-a	Al Deeb, 2020 [64] Case-control	TNF-a was higher in CS and E-Cigs than NS at baseline. A statistically significant reduction from baseline to 12 weeks was reported in the biomarker levels for all the study groups.	Smoke increases inflammation
TNF-a	Al-Aali, 2018 [51] Case-control]	TNF-a (*p* < 0.001) was statistically significantly higher in E-Cigs than NS	Smoke increases gingival inflammation
TNF-a	Alqahtani, 2019 [62] Cross-sectional	TNF-α was significantly higher in CS, WS, and E-Cigs than NS. No statistical differences for cytokines were observed among CS and WS	Smoke increases gingival inflammation
COTININE	Alqahtani, 2019 [62] Cross-sectional	PISF and cotinine levels were significantly higher in CS (*p* < 0.05) and WS (*p* < 0.05) and E-Cigs (*p* < 0.05) than NS.	Smoke increases gingival inflammation
PISF (peri-implant sulcular fluid)	Sinha, 2020 [63] Case-control	PISF concentrations were found relatively higher in E-Cigs than NS	Smoke increases gingival inflammation
PISF	Alqahtani, 2019 [62] Cross-sectional	PISF is significantly higher among smokers than NS	Smoke increases gingival inflammation
PISF	Alqahtani, 2019 [62] Cross-sectional	PISF is higher among smokers	Smoke increases gingival inflammation
PISF	Al-Aali, 2018 [51] Case-control	The PISF volume (*p* < 0.05) collected for E-Cigs was statistically significantly higher than NS	Smoke increases gingival inflammation
PISF	ArRejaie, 2018 [60] Case-control	The PISF volume (*p* < 0.01) collected for CS and vaping individuals was statistically significantly higher than NS	Smoke increases gingival inflammation
PISF	Al Deeb, 2020 [64] Case-control	A statistically significant reduction from baseline to 12 weeks was reported in the biomarker levels for all the study groups.	A statistically significant reduction was reported in the biomarker levels for all the study groups.
MMP-8	Al Deeb, 2020 [64] Case-control	MMP-8 was higher in CS and ES than NS at baseline. A statistically significant reduction from baseline to 12 weeks was reported in the biomarker levels for all the study groups	Smoke increases inflammation
MMP-8	AlJasser, 2021 [65] Case-control	The comparison of mean values of MMP-8, has shown statistically significant change across the three groups over the four intervals of observation (*p* < 0.0001)	Smoke increases inflammation
MMP-9	ArRejaie, 2018 [60] Case-control	MMP-9 (*p* = 0.0198) levels were statistically significantly higher in CS than E-Cigs and NS MMP-9 (*p* = 0.0198) levels were statistically significantly higher in E-Cigs than NS	Smoke increases inflammation

**Table 7:** Risk of bias of the studies included in the systematic review. Response options were: Yes (Y), Probably yes (PY), Probably no (PN), No (N) and No information (NI); “Y” indicates low risk of bias, “PY” indicates a moderate risk of bias; “PN” indicates a serious risk, “N” indicates a critical risk of bias and “NI” indicates no information, as per the ROBINS-I tool.

Study	Bias Due to Confounding	Bias in Selection of Participants	Bias in Measurement Classification of Interventions	Bias Due to Deviations from Intended Interventions	Bias Due to Missing Data	Bias in Measurement of Outcomes	Bias Due to Selection of the Reported Result
Mokeem [50]	Y/PY/ PN/N	Y/PY/ PN/N/NI	Y/PY/ PN/N/NI	Y/PY/ PN/N/NI	Y/PY/ PN/N/NI	Y/PY/ PN/N/NI	Y/PY/ PN/N/NI
Al-Aali [51]	Y/PY/ PN/N	Y/PY/ PN/N/NI	Y/PY/ PN/N/NI	Y/PY/ PN/N/NI	Y/PY/ PN/N/NI	Y/PY/ PN/N/NI	Y/PY/ PN/N/NI
Karaaslan [52]	Y/PY/ PN/N	Y/PY/ PN/N/NI	Y/PY/ PN/N/NI	Y/PY/ PN/N/NI	Y/PY/ PN/N/NI	Y/PY/ PN/N/NI	Y/PY/ PN/N/NI
AlQahtani [53]	Y/PY/ PN/N	Y/PY/ PN/N/NI	Y/PY/ PN/N/NI	Y/PY/ PN/N/NI	Y/PY/ PN/N/NI	Y/PY/ PN/N/NI	Y/PY/ PN/N/NI
BinShabaib [54]	Y/PY/ PN/N	Y/PY/ PN/N/NI	Y/PY/ PN/N/NI	Y/PY/ PN/N/NI	Y/PY/ PN/N/NI	Y/PY/ PN/N/NI	Y/PY/ PN/N/NI
Vohra [55]	Y/PY/ PN/N	Y/PY/ PN/N/NI	Y/PY/ PN/N/NI	Y/PY/ PN/N/NI	Y/PY/ PN/N/NI	Y/PY/ PN/N/NI	Y/PY/ PN/N/NI
Javed [56]	Y/PY/ PN/N	Y/PY/ PN/N/NI	Y/PY/ PN/N/NI	Y/PY/ PN/N/NI	Y/PY/ PN/N/NI	Y/PY/ PN/N/NI	Y/PY/ PN/N/NI
Jeong [26]	Y/PY/ PN/N	Y/PY/ PN/N/NI	Y/PY/ PN/N/NI	Y/PY/ PN/N/NI	Y/PY/ PN/N/NI	Y/PY/ PN/N/NI	Y/PY/ PN/N/NI
Aldakheel [57]	Y/PY/ PN/N	Y/PY/ PN/N/NI	Y/PY/ PN/N/NI	Y/PY/ PN/N/NI	Y/PY/ PN/N/NI	Y/PY/ PN/N/NI	Y/PY/ PN/N/NI
Ibraheem [58]	Y/PY/ PN/N	Y/PY/ PN/N/NI	Y/PY/ PN/N/NI	Y/PY/ PN/N/NI	Y/PY/ PN/N/NI	Y/PY/ PN/N/NI	Y/PY/ PN/N/NI
ALHarthi [59]	Y/PY/ PN/N	Y/PY/ PN/N/NI	Y/PY/ PN/N/NI	Y/PY/ PN/N/NI	Y/PY/ PN/N/NI	Y/PY/ PN/N/NI	Y/PY/ PN/N/NI
ArRejaie [60]	Y/PY/ PN/N	Y/PY/ PN/N/NI	Y/PY/ PN/N/NI	Y/PY/ PN/N/NI	Y/PY/ PN/N/NI	Y/PY/ PN/N/NI	Y/PY/ PN/N/NI
Al-Hamoudi [61]	Y/PY/ PN/N	Y/PY/ PN/N/NI	Y/PY/ PN/N/NI	Y/PY/ PN/N/NI	Y/PY/ PN/N/NI	Y/PY/ PN/N/NI	Y/PY/ PN/N/NI
Alqahtani [62]	Y/PY/ PN/N	Y/PY/ PN/N/NI	Y/PY/ PN/N/NI	Y/PY/ PN/N/NI	Y/PY/ PN/N/NI	Y/PY/ PN/N/NI	Y/PY/ PN/N/NI
Sinha [63]	Y/PY/ PN/N	Y/PY/ PN/N/NI	Y/PY/ PN/N/NI	Y/PY/ PN/N/NI	Y/PY/ PN/N/NI	Y/PY/ PN/N/NI	Y/PY/ PN/N/NI
Al Deeb [64]	Y/PY/ PN/N	Y/PY/ PN/N/NI	Y/PY/ PN/N/NI	Y/PY/ PN/N/NI	Y/PY/ PN/N/NI	Y/PY/ PN/N/NI	Y/PY/ PN/N/NI
AlJasser [65]	Y/PY/ PN/N	Y/PY/ PN/N/NI	Y/PY/ PN/N/NI	Y/PY/ PN/N/NI	Y/PY/ PN/N/NI	Y/PY/ PN/N/NI	Y/PY/ PN/N/NI
Fangxi Xu [66]	Y/PY/ PN/N	Y/PY/ PN/N/NI	Y/PY/ PN/N/NI	Y/PY/ PN/N/NI	Y/PY/ PN/N/NI	Y/PY/ PN/N/NI	Y/PY/ PN/N/NI
Risk of bias judgements	CRITICAL	SERIOUS	LOW	LOW	LOW	MODERATE	LOW

## Data Availability

Medline/PubMed and Cochrane databases.

## References

[1] Tonetti M.S., Greenwell H., Kornman K.S. (2018). Staging and grading of periodontitis: Framework and proposal of a new classification and case definition. J. Periodontol..

[2] Sbordone C., Toti P., Brevi B., Martuscelli R., Sbordone L., Di Spirito F. (2018). Computed tomography-aided descriptive analysis of maxillary and mandibular atrophies. J. Stomatol. Oral Maxillofac. Surg..

[3] Di Spirito F., Toti P., Brevi B., Martuscelli R., Sbordone L., Sbordone C. (2019). Computed tomography evaluation of jaw atrophies before and after surgical bone augmentation. Int. J. Clin. Dent..

[4] Checchi V., Gasparro R., Pistilli R., Canullo L., Felice P. (2019). Clinical Classification of Bone Augmentation Procedure Failures in the Atrophic Anterior Maxillae: Esthetic Consequences and Treatment Options. BioMed Res. Int..

[5] Renvert S., Persson G.R., Pirih F.Q., Camargo P.M. (2018). Peri-implant health, peri-implant mucositis, and peri-implantitis: Case definitions and diagnostic considerations. J. Periodontol..

[6] Di Spirito F., La Rocca M., De Bernardo M., Rosa N., Sbordone C., Sbordone L. (2020). Possible Association of Periodontal Disease and Macular Degeneration: A Case-Control Study. Dent. J..

[7] Chapple I.L.C., Genco R. (2013). On behalf of working group 2 of the joint EFP/AAP workshop. Diabetes and periodontal diseases: Consensus report of the Joint EFP/AAPWorkshop on Periodontitis and Systemic Diseases. J. Periodontol..

[8] Di Spirito F., Schiavo L., Pilone V., Lanza A., Sbordone L., D’Ambrosio F. (2021). Periodontal and Peri-Implant Diseases and Systemically Administered Statins: A Systematic Review. Dent. J..

[9] Genco R.J., Borgnakke W.S. (2013). Risk factors for periodontal disease. Periodontology 2000.

[10] Di Spirito F., Sbordone L., Pilone V., D’Ambrosio F. (2019). Obesity and Periodontal Disease: A Narrative Review on Current Evidence and Putative Molecular Links. Open Dent. J..

[11] Di Spirito F., Toti P., Pilone V., Carinci F., Lauritano D., Sbordone L. (2020). The Association between Periodontitis and Human Colorectal Cancer: Genetic and Pathogenic Linkage. Life.

[12] D’Ambrosio F., Caggiano M., Schiavo L., Savarese G., Carpinelli L., Amato A., Iandolo A. (2022). Chronic Stress and Depression in Periodontitis and Peri-Implantitis: A Narrative Review on Neurobiological, Neurobehavioral and Immune–Microbiome Interplays and Clinical Management Implications. Dent. J..

[13] Chrcanovic B.R., Albrektsson T., Wennerberg A. (2015). Smoking and dental implants: A systematic review and meta-analysis. J. Dent..

[14] Ramaglia L., Di Spirito F., Sirignano M., La Rocca M., Esposito U., Sbordone L. (2019). A 5-year longitudinal cohort study on crown to implant ratio effect on marginal bone level in single implants. Clin. Implant Dent. Relat. Res..

[15] Ramôa C.P., Eissenberg T., Sahingur S.E. (2017). Increasing popularity of waterpipe tobacco smoking and electronic cigarette use: Implications for oral healthcare. J. Periodontal. Res..

[16] Choi K., Forster J.L. (2014). Beliefs and experimentation with electronic cigarettes: A prospective analysis among young adults. Am. J. Prev. Med..

[17] Ratajczak A., Jankowski P., Strus P., Feleszko W. (2020). Heat Not Burn Tobacco Product-A New Global Trend: Impact of Heat-Not-Burn Tobacco Products on Public Health, a Systematic Review. Int. J. Environ. Res. Public Health.

[18] Etter J.-F., Bullen C., Flouris A., Laugesen M., Eissenberg T. (2011). Electronic nicotine delivery systems: A research agenda. Tob. Control.

[19] Cobb N., Abrams D.B. (2011). E-Cigarette or Drug-Delivery Device? Regulating Novel Nicotine Products. N. Engl. J. Med..

[20] Mishra V.K., Kim K.-H., Samaddar P., Kumar S., Aggarwal M., Chacko K. (2017). Review on metallic components released due to the use of electronic cigarettes. Environ. Eng. Res..

[21] Gaur S., Agnihotri R. (2019). Health Effects of Trace Metals in Electronic Cigarette Aerosols-a Systematic Revew. Biol. Trace Elem. Res..

[22] Ralho A., Coelho A., Ribeiro M., Paula A., Amaro I., Sousa J., Marto C., Ferreira M., Carrilho E. (2019). Effects of Electronic Cigarettes on Oral Cavity: A Systematic Review. J. Evid. Based Dent. Pract..

[23] Figueredo C.A., Abdelhay N., Catunda R., Gibson M.P. (2020). The impact of vaping on periodontitis: A systematic review. Clin. Exp. Dent. Res..

[24] Jeong W., Choi D.W., Kim Y.K., Lee H.J., Lee S.A., Park E.C., Jang S.I. (2020). Associations of electronic and conventional cigarette use with periodontal disease in South Korean adults. J. Periodontol..

[25] Sundar I.K., Javed F., Romanos G.E., Rahman I. (2016). E-cigarettes and flavorings induce inflammatory and pro-senescence responses in oral epithelial cells and periodontal fibroblasts. Oncotarget.

[26] Zarabadipour M., Hosseini S.A.H., Haghdoost-Yazdi H., Aali E., Yusefi P., Mirzadeh M., Piri H. (2022). A study on the correlation between smoking and non-enzymatic antioxidant factors of the saliva of healthy smokers and non-smokers. Braz. Dent. Sci..

[27] Pouly S., Benzimra W.T., Soulan M., Blanc A., Zanetti N., Picavet F., Baker P., Haziza G. (2021). Effect of Switching to the Tobacco Heating System Versus Continued Cigarette Smoking on Chronic Generalized Periodontitis Treatment Outcome: Protocol for a Randomized Controlled Multicenter Study. JMIR Res. Protoc..

[28] Pagano S., Negri P., Coniglio M., Bruscoli S., Di Michele A., Marchetti M.C., Valenti C., Gambelunghe A., Fanasca L., Billi M. (2021). Heat-not-burn tobacco (IQOS), oral fibroblasts and keratinocytes: Cytotoxicity, morphological analysis, apoptosis and cellular cycle. An in vitro study. J. Periodontal. Res..

[29] Liberati A., Altman D.G., Tetzlaff J., Mulrow C., Gotzsche P.C., Ioannidis J.P.A., Clarke M., Devereaux P.J., Kleijnen J., Moher D. (2009). The PRISMA statement for reporting systematic reviews and meta-analyses of studies that evaluate health care interventions: Explanation and elaboration. PLoS Med..

[30] Moher D., Liberati A., Tetzlaff J., Altman D.G., PRISMA Group (2009). Preferred reporting items for systematic reviews and meta-analyses: The PRISMA statement. PLoS Med..

[31] Da Costa Santos C.M., Pimenta C.A.M., Nobre M.R.C. (2007). ThePICOstrategyfor the research question construction and evidence search. Rev. Lat. Am. Enfermagem..

[32] Sterne J.A.C., Hernán M.A., Reeves B.C., Savović J., Berkman N.D., Viswanathan M., Henry D., Altman D.G., Ansari M.T., Boutron I. (2016). ROBINS-I: A tool for assessing risk of bias in non-randomised studies of interventions. BMJ.

[33] Javed F., Kellesarian S.V., Sundar I.K., Romanos G.E., Rahman I. (2017). Recent updates on electronic cigarette aerosol and inhaled nicotine effects on periodontal and pulmonary tissues. Oral Dis..

[34] Shaito A., Saliba J., Husari A., El-Harakeh M., Chhouri H., Hashem Y., Shihadeh A., El-Sabban M. (2017). Electronic Cigarette Smoke Impairs Normal Mesenchymal Stem Cell Differentiation. Sci. Rep..

[35] Vyncke T., De Wolf E., Hoeksema H., Verbelen J., De Coninck P., Buncamper M., Monstrey S., Claes K.E. (2020). Injuries associated with electronic nicotine delivery systems: A systematic review. J. Trauma Acute Care Surg..

[36] Irusa K.F., Vence B., Donovan T. (2020). Potential oral health effects of e-cigarettes and vaping: A review and case reports. J. Esthet. Restor. Dent..

[37] Atuegwu N.C., Perez M.F., Oncken C., Thacker S., Mead E.L., Mortensen E.M. (2019). Association between Regular Electronic Nicotine Product Use and Self-Reported Periodontal Disease Status: Population Assessment of Tobacco and Health Survey. Int. J. Environ. Res. Public Health.

[38] Sancilio S., Gallorini M., Cataldi A., DI Giacomo V. (2015). Cytotoxicity and apoptosis induction by e-cigarette fluids in human gingival fibroblasts. Clin. Oral Investig..

[39] Javed F., Rahman I., Romanos G.E. (2019). Tobacco-product usage as a risk factor for dental implants. Periodontology 2000.

[40] Ganesan S.M., Dabdoub S.M., Nagaraja H.N., Scott M.L., Pamulapati S., Berman M.L., Shields P.G., Wewers M.E., Kumar P.S. (2020). Adverse effects of electronic cigarettes on the disease-naive oral microbiome. Sci. Adv..

[41] Andrikopoulos G., Farsalinos K., Poulas K. (2019). ElectronicNicotineDeliverySystems(ENDS) and TheirRelevanceinOralHealth. Toxics.

[42] Nelson J.M., Cuadra G.A., Palazzolo D.L. (2019). A Comparison of Flavorless Electronic Cigarette-Generated Aerosol and Conventional Cigarette Smoke on the Planktonic Growth of Common Oral Commensal Streptococci. Int. J. Environ. Res. Public Health.

[43] Willershausen I., Wolf T., Weyer V., Sader R., Ghanaati S., Willershausen B. (2014). Influence of E-smoking liquids on human periodontal ligament fibroblasts. Head Face Med..

[44] Rouabhia M., Alanazi H., Park H.J., Gonçalves R.B. (2019). Cigarette Smoke and E-Cigarette Vapor Dysregulate Osteoblast Interaction with Titanium Dental Implant Surface. J. Oral Implant..

[45] Holliday R., Preshaw P.M., Ryan V., Sniehotta F.F., McDonald S., Bauld L., McColl E. (2019). A feasibility study with embedded pilot randomised controlled trial and process evaluation of electronic cigarettes for smoking cessation in patients with periodontitis. Pilot Feasibility Stud..

[46] Zanetti F., Titz B., Sewer A., Sasso G.L., Scotti E., Schlage W.K., Mathis C., Leroy P., Majeed S., Torres L.O. (2017). Comparative systems toxicology analysis of cigarette smoke and aerosol from a candidate modified risk tobacco product in organotypic human gingival epithelial cultures: A 3-day repeated exposure study. Food Chem. Toxicol..

[47] Tatullo M., Gentile S., Paduano F., Santacroce L., Marrelli M. (2016). Crosstalk between oral and general health status in e-smokers. Medicine.

[48] Alqahtani S., Cooper B., Spears C.A., Wright C., Shannahan J. (2020). Electronic nicotine delivery system-induced alterations in oral health via saliva assessment. Exp. Biol. Med..

[49] Al Rifaiy M.Q., Qutub O.A., Alasqah M.N., Al-Sowygh Z.H., Mokeem S.A., Alrahlah A. (2018). Effectiveness of adjunctive antimicrobial photodynamic therapy in reducing peri -implant inflammatory response in individuals vaping electronic cigarettes: A randomized controlled clinical trial. Photodiagn. Photodyn. Ther..

[50] Mokeem S.A., Alasqah M.N., Michelogiannakis D., Al-Kheraif A.A., Romanos G.E., Javed F. (2018). Clinical and radiographic periodontal status and whole salivary cotinine, IL-1β and IL-6 levels in cigarette- and waterpipe- smokers and E-cig users. Environ. Toxicol. Pharmacol..

[51] Al-Aali K.A., Alrabiah M., ArRejaie A.S., Abduljabbar T., Vohra F., Akram Z. (2018). Peri-implant parameters, tumor necrosis factor- alpha, and interleukin-1 beta levels in vaping individuals. Clin. Implant. Dent. Relat. Res..

[52] Karaaslan F., Dikilitaş A., Yiğit U. (2020). The effects of vaping electronic cigarettes on periodontitis. Aust. Dent. J..

[53] AlQahtani M.A., Alayad A.S., Al-Shihri A., Oliveira Bello Correa F., Akram Z. (2018). Clinical peri-implant parameters and inflammatory cyto- kine profile among smokers of cigarette, e-cigarette, and waterpipe. Clin. Implant. Dent. Relat. Res..

[54] Binshabaib M., Alharthi S.S., Akram Z., Khan J., Rahman I., Romanos G.E., Javed F. (2019). Clinical periodontal status and gingival crevicular fluid cytokine profile among cigarette-smokers, electronic-cigarette users and never-smokers. Arch. Oral Biol..

[55] Vohra F., Bukhari I.A., Sheikh S.A., Albaijan R., Naseem M. (2020). Comparison of self-rated oral symptoms and periodontal status among cigarette smokers and individuals using electronic nicotine delivery systems. J. Am. Coll. Health.

[56] Javed F., Abduljabbar T., Vohra F., Malmstrom H., Rahman I., Romanos G.E. (2017). Comparison of Periodontal Parameters and Self-Perceived Oral Symptoms Among Cigarette Smokers, Individuals Vaping Electronic Cigarettes, and Never-Smokers. J. Periodontol..

[57] Aldakheel F.M., Alduraywish S.A., Jhugroo P., Jhugroo C., Divakar D.D. (2020). Quantification of pathogenic bacteria in the subgingival oral biofilm samples collected from cigarette-smokers, individuals using electronic nicotine delivery systems and non-smokers with and without periodontitis. Arch. Oral Biol..

[58] Ibraheem W.I., Fageeh H.I., Preethanath R.S., Alzahrani F.A., Al-Zawawi A.S., Divakar D.D., Al-Kheraif A.A. (2020). Comparison of RANKL and osteoprottegerin levels in the gingival crevicular fluid of young cigarette- and waterpipe- smokers and individuals using electronic nicotine delivery systems. Arch. Oral Biol..

[59] Alharthi S.S., BinShabaib M., Akram Z., Rahman I., Romanos G.E., Javed F. (2018). Impact of cigarette smoking and vaping on the outcome of full-mouth ultrasonic scaling among patients with gingival inflammation: A prospective study. Clin. Oral Investig..

[60] ArRejaie A.S., Al-Aali K.A., Alrabiah M., Vohra F., Mokeem S.A., Basunbul G., Alrahla A., Abduljabbar T. (2018). Pro-inflammatory cytokine levels and peri-implant parameters among cigarette smokers, individuals vaping electronic cigarettes and non-smokers. J. Periodontol..

[61] Al-Hamoudi N., Alsahhaf A., Al Deeb M., Alrabiah M., Vohra F., Abduljabbar T. (2020). Effect of scaling and root planing on the expression of anti-inflammatory cytokines (IL-4, IL-9, IL-10, and IL-13) in the gingival crevicular fluid of electronic cigarette users and non-smokers with moderate chronic periodontitis. J. Periodontal. Implant Sci..

[62] Alqahtani F., Alqahtani M., Shafqat S.S., Akram Z., Al-Kheraif A.A., Javed F. (2019). Efficacy of mechanical debridement with adjunctive probiotic therapy in the treatment of peri-implant mucositis in cigarette-smokers and never-smokers. Clin. Implant Dent. Relat. Res..

[63] Sinha D.K., Kumar A., Khan M., Kumari R., Kesari M. (2020). Vishal Evaluation of tumor necrosis factor-alpha (TNF-α) and interleukin (IL)-1β levels among subjects vaping e-cigarettes and nonsmokers. J. Fam. Med. Prim. Care.

[64] Al Deeb M., Alsahhaf A., Alhamoudi N., Al-Aali K.A., Abduljabbar T. (2020). Clinical and microbiological outcomes of photodynamic and systemic antimicrobial therapy in smokers with peri-implant inflammation. Photodiagn. Photodyn. Ther..

[65] AlJasser R., Zahid M., AlSarhan M., AlOtaibi D., AlOraini S. (2021). The effect of conventional versus electronic cigarette use on treatment outcomes of peri-implant disease. BMC Oral Health.

[66] Xu F., Aboseria E., Janal M.N., Pushalkar S., Bederoff M.V., Vasconcelos R., Sapru S., Paul B., Queiroz E., Makwana S. (2021). Comparative Effects of E-Cigarette Aerosol on Periodontium of Periodontitis Patients. Front. Oral Health.

[67] Bergström J. (2004). Tobaccosmokingand chronic destructive periodontal disease. Odontology.

[68] Dietrich T., Bernimoulin J.P., Glynn R.J. (2004). The effect of cigarette smoking on gingival bleeding. J. Periodontol..

[69] Knight E.T., Liu J., Seymour G., Faggion C.M., Cullinan M. (2016). Risk factors that may modify the innate and adaptive immune responses in periodontal diseases. Periodontology 2000.

[70] Rakic M., Struillou X., Petkovic-Curcin A., Matic S., Canullo L., Sanz M., Vojvodic D. (2014). Estimation of Bone Loss Biomarkers as a Diagnostic Tool for Peri-Implantitis. J. Periodontol..

[71] Petkovic A.B., Matic S.M., Stamatovic N.V., Vojvodic D.V., Todorovic T.M., Lazic Z.R., Kozomara R.J. (2010). Pro-inflammatory cytokines (IL-1beta and TNF-alpha) and chemokines (IL-8 and MIP-1alpha) as markers of peri-implant tissue condition. Int. J. Oral Maxillofac. Surg..

[72] Graves D.T., Li J., Cochran D.L. (2010). Inflammation and Uncoupling as Mechanisms of Periodontal Bone Loss. J. Dent. Res..

[73] Abduljabbar T., Vohra F., Javed F., Akram Z. (2017). Antimicrobial photodynamic therapy adjuvant to non-surgical periodontal therapy in patients with diabetes mellitus: A meta-analysis. Photodiagn. Photodyn. Ther..

[74] Bostanci N., Ilgenli T., Emingil G., Afacan B., Han B., Töz H., Atilla G., Hughes F.J., Belibasakis G.N. (2007). Gingival crevicular fluid levels of RANKL and OPG in periodontal diseases: Implications of their relative ratio. J. Clin. Periodontol..

[75] Teodorescu A.C., Martu I., Teslaru S., Kappenberg-Nitescu D.C., Goriuc A., Luchian I., Martu M.A., Solomon S.M., Mârțu S. (2019). Assessment of Salivary Levels of RANKL and OPG in Aggressive versus Chronic Periodontitis. J. Immunol. Res..

[76] Algate K., Haynes D.R., Bartold P.M., Crotti T.N., Cantley M.D. (2016). The effects of tumour necrosis factor-α on bone cells involved in periodontal alveolar bone loss; osteoclasts, osteoblasts and osteocytes. J. Periodontal. Res..

[77] Reznick A.Z., ECross C., Hu M.L., Suzuki Y.J., Khwaja S., Safadi A., AMotchnik P., Packer L., Halliwell B. (1992). Modification of plasma proteins by cigarette smoke as measured by protein carbonyl formation. Biochem. J..

[78] Epstein F.H., Weiss S.J. (1989). Tissue Destruction by Neutrophils. N. Engl. J. Med..

[79] Di Spirito F., Iacono V.J., Iandolo A., Amato A., Sbordone L. (2021). Evidence-based Recommendations on Periodontal Practice and the Management of Periodontal Patients during and after the COVID-19 Era: Challenging Infectious Diseases Spread by Air-borne Transmission. Open Dent..

[80] Graziani F., Karapetsa D., Alonso B., Herrera D. (2017). Nonsurgical and surgical treatment of periodontitis: How many options for one disease?. Periodontology 2000.

[81] Lauritano D., Moreo G., Carinci F., Della Vella F., Di Spirito F., Sbordone L., Petruzzi M. (2019). Cleaning Efficacy of the XP-Endo^®^ Finisher Instrument Compared to Other Irrigation Activation Procedures: A Systematic Review. Appl. Sci..

[82] Di Spirito F., Pelella S., Argentino S., Sisalli L., Sbordone L. (2022). Oral manifestations and the role of the oral healthcare workers in COVID-19. Oral. Dis..

[83] Di Spirito F., Scelza G., Fornara R., Giordano F., Rosa D., Amato A. (2022). Post-operative endodontic pain management: An overview of systematic reviews on post-operatively administered oral medications and integrated evidence-based clinical recommendations. Healthcare.

[84] Barone A., Chatelain S., Derchi G., Di Spirito F., Martuscelli R., Porzio M., Sbordone L. (2020). Effectiveness of antibiotics in preventing alveolitis after erupted tooth extraction: A retrospective study. Oral Dis..

[85] Pisano M., Amato A., Sammartino P., Iandolo A., Martina S., Caggiano M. (2021). Laser Therapy in the Treatment of Peri-Implantitis: State-of-the-Art, Literature Review and Meta-Analysis. Appl. Sci..

[86] Di Spirito F., Argentino S., Martuscelli R., Sbordone L. (2019). MRONJ incidence after multiple teeth extractions in patients taking oral bisphosphonates without “drug holiday”: A retrospective chart review. Oral Implantol..

